# Etiology and Pathophysiology of Hypoparathyroidism: A Narrative Review

**DOI:** 10.1002/jbmr.4714

**Published:** 2022-11-23

**Authors:** Janice L. Pasieka, Kelly Wentworth, Caitlin T. Yeo, Serge Cremers, David Dempster, Seiji Fukumoto, Ravinder Goswami, Pascal Houillier, Michael A. Levine, Jesse D. Pasternak, Nancy D. Perrier, Antonio Sitges-Serra, Dolores M. Shoback

**Affiliations:** 1 Clinical Professor of Surgery and Oncology, Cumming School of Medicine, University of Calgary, Calgary, AB, Canada; 2 Assistant Adjunct Professor of Medicine, University of California, San Francisco, Zuckerberg San Francisco General Hospital, San Francisco, CA, USA; 3 Clinical Lecturer of Surgery and Oncology, Cumming School of Medicine, University of Calgary, Calgary, AB, Canada; 4 Associate Professor of Pathology and Cell Biology and Medicine, Vagelos College of Physicians and Surgeons of Columbia University, New York, NY, USA; 5 Professor of Clinical Pathology and Cell Biology, Vagelos College of Physicians and Surgeons of Columbia University, New York, NY, USA; 6 Specially Appointed Professor, Fujii Memorial Institute of Medical Sciences, Institute of Advanced Medical Sciences, Tokushima University, Tokushima, Japan; 7 Professor, Department of Endocrinology and Metabolism, All India Institute of Medical Sciences, New Delhi, India; 8 Département de Physiologie, Professor of Physiology, Centre de Recherche des Cordeliers, INSERM, Sorbonne Université, Université de Paris, Assistance Publique-Hôpitaux de Paris, Hôpital Européen Georges Pompidou, Paris, France; 9 Professor Emeritus of Pediatrics and Medicine, University of Pennsylvania, Director, Center for Bone Health, Children’s Hospital of Philadelphia, Philadelphia, PA, USA; 10 Endocrine Surgery Section Head, Division of General Surgery, Department of Surgery, University Health Network, University of Toronto, Toronto, ON, Canada; 11 Professor of Surgery, University of Texas MD Anderson Cancer Center, Department of Surgical Oncology, Section of Surgical Endocrinology, Houston, TX, USA; 12 Emeritus Professor, Universitat Autònoma de Barcelona, Endocrine Surgery, Hospital del Mar, Barcelona, Spain; 13 Professor of Medicine, University of California, San Francisco, Endocrine Research Unit, San Francisco Veterans Affairs Medical Center, San Francisco, CA, USA

**Keywords:** PTH/VIT D/FGF23, CELL/TISSUE SIGNALING, ENDOCRINE PATHWAYS, PARATHYROID-RELATED DISORDERS, DISORDERS OF CALCIUM/PHOSPHATE METABOLISM, HORMONE REPLACEMENT/RECEPTOR MODULATORS, THERAPEUTICS

## Abstract

The approach utilized a systematic review of the medical literature executed with specifically designed criteria that focused on the etiologies and pathogenesis of hypoparathyroidism. Enhanced attention by endocrine surgeons to new knowledge about parathyroid gland viability are reviewed along with the role of intraoperative parathyroid hormone (ioPTH) monitoring during and after neck surgery. Nonsurgical etiologies account for a significant proportion of cases of hypoparathyroidism (~25%), and among them, genetic etiologies are key. Given the pervasive nature of PTH deficiency across multiple organ systems, a detailed review of the skeletal, renal, neuromuscular, and ocular complications is provided. The burden of illness on affected patients and their caregivers contributes to reduced quality of life and social costs for this chronic endocrinopathy. © 2022 The Authors. *Journal of Bone and Mineral Research* published by Wiley Periodicals LLC on behalf of American Society for Bone and Mineral Research (ASBMR).

## Introduction

Parathyroid hormone (PTH) is essential for calcium (Ca) and phosphorus (P) homeostasis through its direct actions on bone resorption, kidney transport of Ca and P, and indirect actions to increase intestinal Ca absorption through enhanced 1,25 (OH)_2_ vitamin D [1,25(OH)_2_ D] production. A rare disorder of impaired or inadequate PTH secretion, hypoparathyroidism, leads to hypocalcemia, hyperphosphatemia, and the clinical consequences described herein. These consequences result from the lack of PTH and the resulting mineral disturbances on multiple tissues.

The estimated prevalence of hypoparathyroidism is ~23 to 37 cases per 100,000 person-years.^(1)^ Approximately 25% of cases arise from genetic or metabolic disorders, autoimmune destruction, or infiltration of the glands (Table 1). Transient hypoparathyroidism and hypocalcemia can also occur with magnesium (Mg) depletion or excess. Many cases remain idiopathic. Most commonly, however, ~75% of cases of hypoparathyroidism are iatrogenic following neck surgery.^(1–4)^

**Fig. 1.**
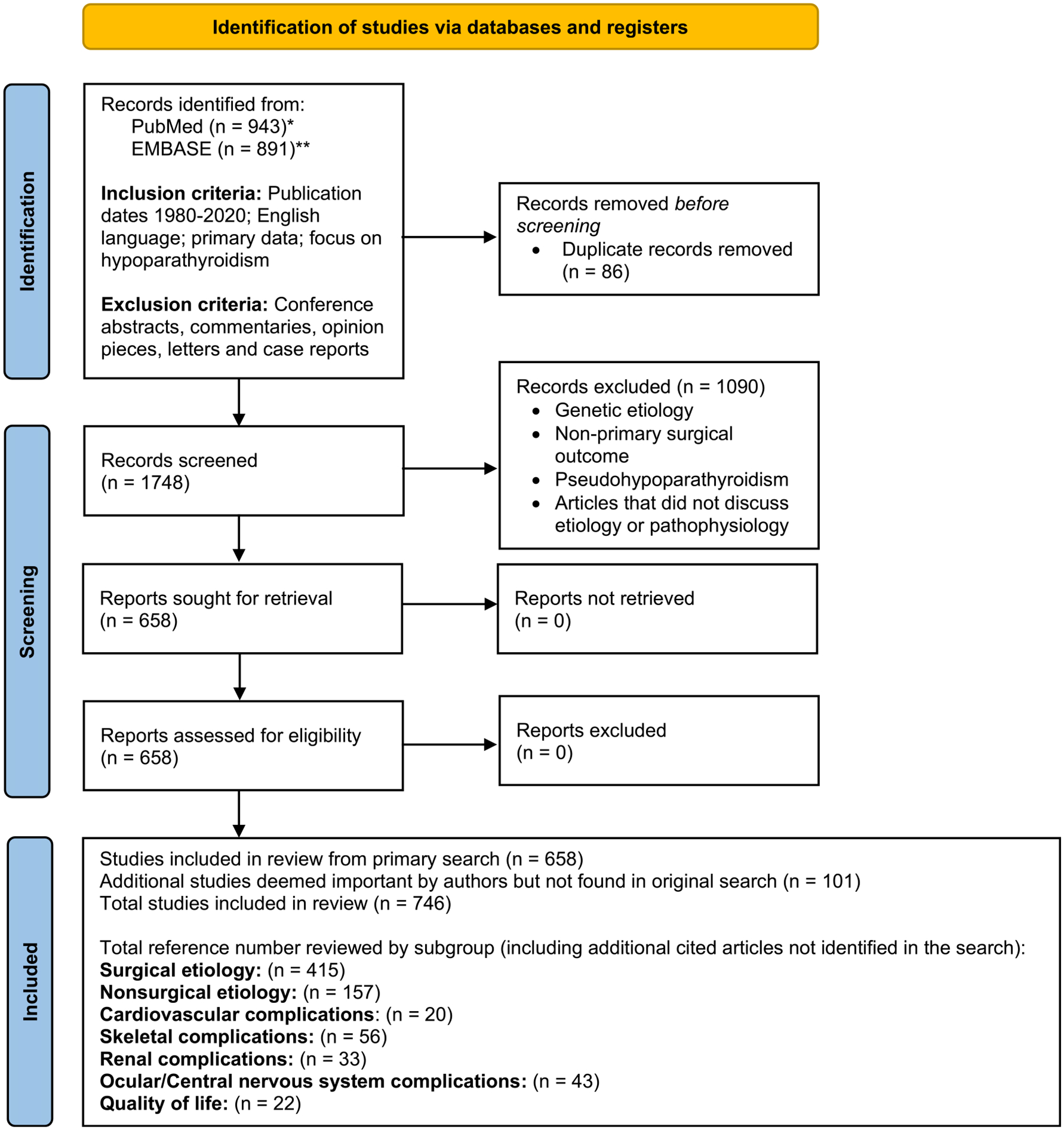
PRISMA 2020 flow diagram for new systematic reviews which included searches of databases and registers only. The search was executed with the following approach: *PubMed Search Terms. (“Hypoparathyroidism/etiology”[Majr] OR “Hypoparathyroidism/pathology”[Majr] OR ((hypoparathyroidism [ti] OR hypoparathyroidism[ot]) AND (etiology OR pathophysiology OR physiopathology OR pathology))) AND (random* OR control* OR cohort OR case–control OR “clinical trial” OR review[pt] OR systematic[sb]) NOT (letter[pt] OR editorial[pt] OR case reports[pt]) AND ((1980:3000/12/12[pdat]). **Embase Search Terms. ((‘hypoparathyroidism’/exp OR hypoparathyroidism) AND (‘pathophysiology’/exp OR pathophysiology OR ‘etiology’/exp OR etiology) AND [humans]/lim AND [english]/lim AND (‘case control study’/de OR ‘clinical article’/de OR ‘clinical study’/de OR ‘clinical trial’/de OR ‘cohort analysis’/de OR ‘comparative study’/de OR ‘controlled clinical trial’/de OR ‘controlled study’/de OR ‘cross sectional study’/de OR ‘human experiment’/de OR ‘longitudinal study’/de OR ‘major clinical study’/de OR ‘multicenter study’/de OR ‘observational study’/de OR ‘prospective study’/de OR ‘randomized controlled trial’/de OR ‘randomized controlled trial topic’/de OR ‘retrospective study’/de OR ‘systematic review’/de) AND [1980–2021]/py) AND (‘article’/it OR ‘article in press’/it). From: Page and colleagues.^(5)^ For more information, visit: http://www.prisma-statement.org/.

This systematic review was prepared in consultation with an experienced medical librarian who executed a comprehensive search in PubMed and Embase. The complete search strings are supplied in Fig. 1. All articles from the database search were added to a reference manager, and duplicates were removed. Inclusion and exclusion criteria were developed to screen articles by abstract using two independent reviewers (KW and CY). Inclusion criteria included articles with publication dates from 1980 to 2020, in the English language, and with primary data and a focus on etiology and pathophysiology of hypoparathyroidism. Exclusion criteria included any article not primarily focusing on the etiology or pathophysiology of hypoparathyroidism, and those with a primary focus on genetic etiologies of hypoparathyroidism were excluded because that topic is covered elsewhere. Conference abstracts, opinion pieces, commentaries, letters, and case reports were excluded. The full text of articles selected by title and abstract screening were reviewed using the same criteria and methods. Articles retained for analysis underwent cited reference searching. Articles from cited reference searching and those recommended by experts were included if they met the above inclusion and exclusion criteria. Results are presented in a Preferred Reporting Items for Systematic reviews and Meta-Analyses (PRISMA) flow diagram and articles were further categorized according to subtopic (Fig. 1). A meta-analysis was not performed given the noninterventional nature of the data collected. Risk-of-bias (RoB) and quality assessment were not performed given the nature of the question and data collected.

## Etiology of Hypoparathyroidism

### Postsurgical hypoparathyroidism

Postsurgical hypoparathyroidism occurs after inadvertent removal or devascularization of the parathyroid glands during surgery. Routine deliberate removal of glands for autotransplantation has also been associated with a higher risk of postsurgical hypoparathyroidism.^(6,7)^) Postoperative hypocalcemia is the most common complication following bilateral thyroid surgery, reoperative thyroid and parathyroid surgeries, and extensive neck dissections. The estimated prevalence of temporary and permanent hypoparathyroidism varies widely from 14% to 43% and 1% to 25%, respectively.^(6,8–13)^ Much of the variation stems from the lack of a single definition of postsurgical hypoparathyroidism, variable timing of biochemical monitoring, incomplete follow-up, different postoperative policies for Ca and vitamin D supplementation, single institutional/surgeon retrospective series, and lack of corroborating intact PTH (iPTH) values.^(14–16)^ Growing evidence from large population cohorts and national audits indicate that permanent hypoparathyroidism is a significant clinical problem.

Crosslinking data from the Swedish Drug Register and all patients who underwent bilateral thyroidectomy for benign disease, Anneback and colleagues^(9)^ estimated that 12.5% of patients remained hypoparathyroid 1 year following surgery. Utilizing National Surgical Quality Improvement Program (NSQIP) data, Kazaure and colleagues^(17)^ found that 5.8% (428/7366) of patients experienced severe hypocalcemia post-thyroidectomy (defined as readmission, utilization of intravenous Ca, or emergent postoperative clinic visits within 30 days of the index operation), illustrating the magnitude of this problem and lack of standardized guidelines for management of post-thyroidectomy hypocalcemia. Emerging data indicate that this surgical complication has significant long-term morbidity and mortality affecting renal, cardiovascular, and potentially cancer events,^(18–20)^ the details of which are discussed by Task Force 1.^(21)^ These data underscore the importance of proactive preservation of the parathyroid glands and their vascular supply during thyroid and parathyroid surgery.

### Definition of postsurgical hypoparathyroidism

#### Immediate postsurgical hypoparathyroidism

**Fig. 2.**
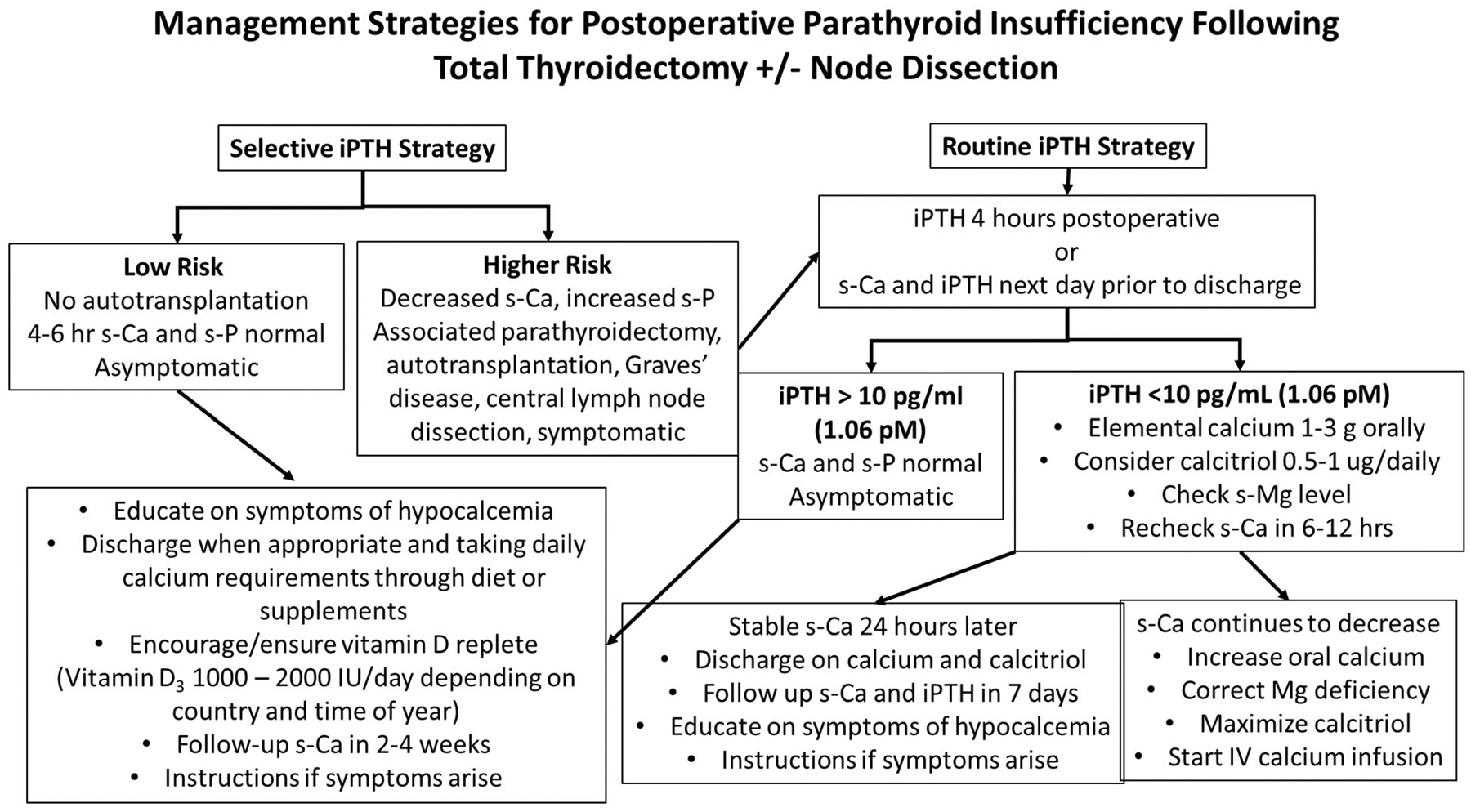
An algorithm for management strategies for the evaluation and treatment of postoperative parathyroid gland insufficiency following total thyroidectomy with or without lymph node dissection.

Immediate postsurgical hypoparathyroidism is defined as a serum albumin-adjusted calcium (s-Ca) level that is <2 mmol/L (8.0 mg/dL), with or without symptoms. It occurs frequently in patients following thyroid surgery and is usually transient (>85% to 90% of the time). A patient’s s-Ca and serum P (s-P) should be monitored within the first 6 hours postoperatively and treatment initiated prior to the onset of symptoms.^(22)^ Symptomatic patients and patients in whom postsurgical parathyroid failure is suspected should receive therapy with Ca salts and activated vitamin D metabolites prior to discharge. Patients who undergo parathyroid/thyroid surgery should have normal serum concentrations of 25(OH) vitamin D (>50 nmol/L or 20 ng/mL) and receive supplementation if levels are lower than this. The surgical procedure and the number of glands remaining in situ can predict the likelihood of immediate, protracted and permanent postoperative parathyroid failure.^(11,23)^ A rising s-P in conjunction with a declining s-Ca following a total thyroidectomy suggests inadequate PTH secretion and is a simple, cost-effective screening tool in lieu of intact PTH (iPTH) measurements, to delineate those at risk of developing hypocalcemia.^(22)^ Many centers utilize iPTH measurements within 6 hours postoperatively or prior to discharge and institute calcium and activated vitamin D metabolite therapy if iPTH drops below 10 pg/mL (1.06pM)^(4,11,24–27)^ (Fig. 2) to prevent both biochemical and clinical hypocalcemia. Although some authors and guidelines advocate for routine Ca supplementation as a cost-effective strategy in all postoperative patients,^(23,28–30)^ in a recent Cochrane review, there was no high-quality evidence to support this approach,^(31)^ and numerous reports have illustrated the danger of unchecked Ca supplementation, poor patient compliance, and medication side-effects.^(17,30,32)^

Resolution of parathyroid insufficiency occurs in the majority (70% to 80%) of patients within 1 month following surgery.^(11,24,33)^ Patients who still require supplement therapy due to persistently low or absent iPTH after 1 month are considered to have *protracted postsurgical hypoparathyroidism*. The prevalence of protracted postsurgical hypoparathyroidism varies from 13% to 44%.^(11,14,16,34)^ Others have demonstrated that the rate of protracted postsurgical hypoparathyroidism depends on the number of glands left in situ, ranging from 13% to 15% when all four glands remain in situ, to 40% to 44% when two or fewer remain in situ.^(11,23)^ The probability of recovering parathyroid function over the next 12 months is approximately 75%.

There is lack of consensus on the definition of *permanent postsurgical hypoparathyroidism*.^(2,8,16)^ Most investigators and guidelines use the need for Ca salts and active vitamin D therapy at 6 or 12 months.^(35)^ Permanent postsurgical hypoparathyroidism includes three distinct subcategories (Table 2). Approximately 10% to 15% of cases recover after 1 year.^(14,24)^

### Risk factors for surgical hypoparathyroidism

The parathyroid glands are at risk during thyroid surgery due to their small size, close proximity to the thyroid gland, and delicate blood supply. Devascularization and/or resection of these glands increase the risk of both transient and permanent postsurgical hypoparathyroidism (odds ratio [OR] 2.79, OR 4.62, respectively).^(36)^ Hypoparathyroidism can lead to acute postoperative complications (Table 3), hospital readmission,^(17,37,38)^ significant long-term morbidity, and increased mortality (adjusted hazard ratio [HR] 2.09).^(18,20)^ Risk factors for the development of hypoparathyroidism can be divided into patient, disease, and operative factors (Table 4).

A meta-analysis of 115 observational studies identified preoperative vitamin D deficiency, Graves’ disease (OR 1.75), and inadvertent parathyroidectomy (parathyroids found in the pathology specimen not intended to be removed during surgery) (OR 1.90) as risk factors for postoperative hypocalcemia.^(6)^ Bai and colleagues^(39)^ found an average rate of incidental parathyroidectomy of 12.4%. Risk factors for incidental parathyroidectomy include central neck dissection (relative risk [RR] 2.35), reoperation (RR 1.81), malignancy (RR 1.60), and total thyroidectomy (RR 1.42). Incidental parathyroidectomy increases the risk of symptomatic hypocalcemia (RR 1.54), with permanent hypoparathyroidism occurring at a rate 6.7% in patients with versus 2.2% in those without incidental parathyroidectomy (RR 3.10).

Surgical experience, expertise and technique play key roles in determining the risk of hypoparathyroidism.^(6,10,36,40,41)^ A recent single-center study of >1000 thyroidectomies identified an average inadvertent parathyroidectomy rate of 22.4% (range, 16.9% to 43.6%) directly correlating with surgeon volume (*R*^2^ = 0.77 for thyroidectomies and *R*^2^ = 0.93 for central neck dissection).^(36)^ In their multivariate analysis, low-volume surgeon (OR 2.94), extrathyroidal extension of the tumor (OR 3.13), and prophylactic (OR 2.68) or therapeutic central neck dissection (OR 4.44) were associated with incidental parathyroidectomy.

Surgical risk factors for hypoparathyroidism in children are less well known, due to the small number of studies and cohort sizes, but they are similar to those in adults.^(42–45)^ Pediatric patients present unique challenges for the surgeon due to small gland size and appearance of brown fat, thymus, and lymph nodes.

### Role of parathyroid autotransplantation

Autotransplantation of parathyroid glands initially gained popularity in the treatment of parathyroid hyperplasia following total parathyroidectomy.^(46–48)^ This evolved into routine autotransplantation of normal parathyroid glands during thyroid surgery in an attempt to reduce postsurgical hypoparathyroidism.^(47,48)^ Several authors have demonstrated biochemical graft uptake in 83% to 90% of patients, even when normal parathyroid glands were left in situ.^(49,50)^ This led some surgeons to recommend routine or liberal autotransplantation of at least one parathyroid gland during thyroid surgery.^(51–53)^ However, a recent meta-analysis of 25 studies showed an increased risk of postsurgical hypoparathyroidism in thyroidectomy patients who underwent parathyroid autotransplantation, and the number of autotransplanted glands correlated positively with the rate of postsurgical hypoparathyroidism.^(7)^ This analysis included both liberal autotransplantation and its use only after incidental parathyroidectomy. Thus, autotransplantation is likely not the dominant risk factor for postsurgical hypoparathyroidism, but rather the direct result of removal and/or devascularization of the gland(s).

Lorente-Poch and colleagues^(23)^ followed 657 total thyroidectomy patients and demonstrated that the rates of immediate, protracted, and permanent postsurgical hypoparathyroidism were significantly lower in those patients in whom all four parathyroid glands were left in situ, compared to those with three or fewer glands in situ, illustrating the importance of in situ preservation of all the glands.^(23)^ Autotransplantation should only be utilized when inadvertent parathyroidectomy has occurred, and every effort should be made to preserve the parathyroid glands in situ.^(11,23,54,55)^

Studies have shown that intraoperative identification of parathyroid glands results in a lower incidence of inadvertent parathyroidectomy.^(11,23,56,57)^ Discoloration of three or more glands is predictive of transient hypoparathyroidism, but challenges remain in determining the viability of glands that appear normal to the eye.^(58,59)^ Macroscopic appearance of the parathyroid gland has a sensitivity of only 78% for predicting postoperative hypocalcemia.^(30)^ Even those glands that appear abnormal may retain function. Rudin and colleagues^(60)^ demonstrated that visual inspection overestimated parathyroid ischemia compared to indocyanine green (ICG)-labeled blood-flow, illustrating in many cases autotransplantation would remove functioning glands if based solely on the gross appearance of the gland.

### Emerging technology and intraoperative assessment of parathyroid viability

ICG is a water-soluble, low molecular weight, cyanine-based dye that is rapidly bound to plasma proteins after intravenous injection. ICG has recently been utilized to assess the viability of parathyroid glands. In a randomized trial of 146 patients with least one ICG well-perfused parathyroid gland demonstrated at the time of surgery, postoperative normocalcemia could be predicted.^(61,62)^ In addition to ICG, other technologies such as Laser Speckle Contrast Imaging,^(63)^ have been proposed to help assess the parathyroids. Near-infrared autofluorescence, which does not require injected dye for parathyroid visualization, is also considered an evolving technology in this field.^(64)^ However, surgical technique is the main factor preventing the devascularization of the glands or incidental parathyroidectomy. Although several imaging modalities have been studied to help prevent incidental parathyroidectomy, few have evaluated clinically relevant end-points such as surgical hypoparathyroidism.^(65–69)^

### iPTH for diagnosis and management of acute surgical hypoparathyroidism

The optimal strategy for identifying patients who will develop postsurgical hypoparathyroidism has yet to be defined.^(30)^ Same-day discharge and limited healthcare resources have resulted in a variety of Ca management strategies following thyroidectomy.^(70)^ Kazaure and colleagues^(17)^ found that 6% of thyroidectomy patients in the United States developed severe hypocalcemia following discharge, illustrating the need for standardized guidelines and policies for postoperative hypocalcemia management. The utility of iPTH-based protocols is evolving and should be utilized to identify those patients at risk for developing parathyroid insufficiency prior to discharge from hospital,^(71)^ keeping in mind that studies have found the utilization of iPTH criteria are not as reliable in vitamin D–deficient patients^(72,73)^ (Fig. 2).

There is no consensus regarding the timing or cutoff values for iPTH measurements in postoperative stratification of patients following thyroidectomy. Some authors obtain iPTH while closing the skin,^(74,75)^ others at 4 hours^(25–27,76,77)^ to select patients for early replacement therapy and safe same-day discharge strategies, and others the following morning.^(76)^ Surgeons need to adopt a consistent algorithmic strategy for management of patients following total thyroidectomy that is in keeping with their health resources and discharge and follow-up practice (Fig. 2). In general, medical therapy (2 to 3 g of elemental Ca per day and 0.5 to 1.5 μg calcitriol per day) should be started if iPTH concentrations drop below 10 pg/mL (1.06pM) or decline by more than 70% of the preoperative values at 4 hours after surgery.^(25–27,76)^

Between 70% to 80% of cases of postoperative parathyroid failure will recover within 1 month after thyroidectomy.^(11,78)^ Serum Ca, P, and iPTH should be checked within 2 weeks to determine whether parathyroid function has recovered and replacement therapy can be terminated. The chances of parathyroid recovery are dependent on the number of parathyroid glands remaining in situ and s-Ca and iPTH levels. Detectable iPTH, four glands left in situ, and serum Ca >2.25 mmol/L (9.00 mg/dL) at 1 month after surgery are favorable predictors of recovery.^(11,23,78–81)^

Follow-up strategies beyond 1 postoperative month of protracted hypoparathyroidism are important, as many patients will recover over the course of the year (75%), and up to 12% beyond 1 year.^(14,23,24,82)^ Monthly iPTH and s-Ca measurements for at least 12 months are suggested, to diagnose persistence of hypoparathyroidism or recovery of the parathyroid function.

### Nonsurgical etiologies of hypoparathyroidism

Approximately 25% of adults with hypoparathyroidism have a developmental, genetic, autoimmune, metabolic, or environmental condition that impairs either the secretion or action of PTH or alters parathyroid gland mass (Table 1).^(1,3,83)^ Medical hypoparathyroidism is particularly important in pediatric patients. Functional hypoparathyroidism can be broadly categorized as a condition in which hypocalcemia and hyperphosphatemia are the consequence of a disorder that (i) impairs development or survival of parathyroid glands, (ii) decreases secretion of biologically active PTH, or (iii) reduces target organ responsiveness to PTH (pseudohypoparathyroidism).

#### Genetic disorders

The genetic disorders that are associated with hypoparathyroidism are discussed more fully elsewhere (see manuscript for Task Force 3^(35)^).

#### Autoimmune hypoparathyroidism

Autoimmune hypoparathyroidism can occur as an isolated endocrinopathy or as a part of the autoimmune polyglandular syndrome type I (APS-1), a genetic disorder caused by mutations in the autoimmune regulator (*AIRE)* gene. APS-1 is also known as autoimmune polyendocrinopathy candidiasis ectodermal dystrophy (APECED).^(84,90–92)^

In APS-1, tissue-specific autoantibodies targeted against the parathyroid, thyroid, and adrenal glands have been identified and support an autoimmune etiology. Key studies have identified anticytokine antibodies that are highly specific and sensitive markers for APS-1, including autoantibodies directed against interferon (IFN)-ω, IFN-α2A, interleukin (IL)-17F, and IL-22.^(93)^ NACHT leucine-rich repeat protein 5 (NALP5) has been identified as a target antigen for autoimmune attack in the parathyroid cells, leading to tissue destruction. Isolated autoimmune hypoparathyroidism has also been described in adult patients who develop circulating antibodies targeted against the extracellular domain of the calcium-sensing receptor (CaSR).^(93)^ These antibodies activate the receptor and thereby inhibit PTH secretion.^(94–96)^ Patients with noncytotoxic anti-CaSR antibodies may recover from hypoparathyroidism over time as antibody titers decrease.

#### Radiation, toxins, and medications

Ionizing radiation can have dichotomous effects on parathyroid tissue. High-dose radioactive iodine that is administered for the treatment of thyroid cancer has been associated with hypoparathyroidism,^(97,98)^ but this is a rare event. Similarly, high-dose external beam radiation has been linked to parathyroid damage. At lower levels of exposure, ionizing radiation has also been shown to induce parathyroid adenomas and primary hyperparathyroidism. Deposition of iron, copper, or aluminum in parathyroid tissue, as can be seen in hemochromatosis or transfusion dependence, Wilson’s disease, or renal dysfunction (with use of aluminum-containing phosphate binders), can lead to destruction of the glands.^(1,3,83)^ In addition, invasion of the parathyroid glands by neoplastic, granulomatous, or inflammatory cells (eg, sarcoidosis, Riedel struma, or human immunodeficiency virus [HIV]) or infiltration by amyloid protein can also damage the parathyroid glands.^(83,99)^

Very few medications or toxins can damage the parathyroid glands. Most notable are l-asparaginase, which is used in the treatment of leukemia,^(100)^ and nivolumab, an anti-PD-1 immune checkpoint inhibitor that has been associated with development of activating autoantibodies against the CaSR.^(101)^ In addition, fetal exposure to retinoids, alcohol, or hyperglycemia via poorly controlled gestational diabetes are associated with a DiGeorge-like syndrome and parathyroid dysgenesis without an obvious genetic deletion, although clinical hypoparathyroidism is uncommon.^(102)^

#### Metabolic disorders

Children with severe burns can develop hypocalcemia within days after burn injury, irrespective of parenteral Ca supplementation, and urinary Ca excretion is typically elevated. Serum concentrations of PTH are inappropriately low for the blood Ca levels, indicating that children with burns have not only hypocalcemia and hypercalciuria but also hypoparathyroidism.^(103)^ Although these children have severe magnesium (Mg) depletion, replacement with supplemental Mg does not reverse the parathyroid defect, which has been attributed to upregulation of the CASR through an as yet unknown mechanism.^(104)^ An increase in circulating levels of IL-1β and IL-6 have been proposed, as in vitro studies have shown that these cytokines are capable of upregulating the parathyroid gland CASR.

Hypoparathyroidism can also be caused by either hypermagnesemia or hypomagnesemia. Hypomagnesemia is associated with impaired release of PTH from the parathyroid cell, likely through a disturbance in the stimulus-secretion coupling mechanism.^(105)^ Because both Mg and Ca activate the CaSR and reduce PTH synthesis and secretion,^(106)^ albeit with different potencies, elevated levels of extracellular Mg can lead to functional hypoparathyroidism. Hypomagnesemia can also cause PTH resistance and functional hypoparathyroidism.^(105,107–109)^

#### Maternal hypercalcemia

Infants exposed in utero to maternal hypercalcemia (eg, in the context of maternal primary hyperparathyroidism, vitamin D disorders, or maternal familial hypocalciuric hypercalcemia with a nonaffected fetus) are at risk of suppression of parathyroid function and postnatal hypocalcemia. Additional risks to the developing fetus include intrauterine growth retardation, preterm delivery, and intrauterine fetal death if the hyperparathyroidism in the mother remains untreated.^(110–112)^

#### Idiopathic

Hypoparathyroidism is considered to be idiopathic when extensive evaluation of all potential causes of hypoparathyroidism fail to disclose an etiology.^(113)^ It is conceivable that many patients with early-onset or congenital hypoparathyroidism will have underlying genetic etiologies that may require the application of unbiased molecular genetic technologies, such as whole-exome sequencing or whole-genome sequencing, to diagnose. In contrast, individuals with late-onset hypoparathyroidism may have an underlying autoimmune etiology. In the absence of a clear etiology for hypoparathyroidism, these subjects should be monitored closely for the development of additional disorders.

## Pathophysiology of the Disease and Selected Complications

### Renal manifestations

The kidney is a target organ often damaged in patients with hypoparathyroidism. Under normal conditions, PTH is critical to sustain renal distal tubular reabsorption of filtered Ca and, therefore, to maintain the extracellular Ca concentrations. When PTH is absent, less Ca is reabsorbed, and the extracellular Ca level is not maintained within the normal range. However, the absolute amount of urinary Ca is not usually high because there is a reduced filtered load in untreated patients. Similarly, insufficient PTH secretion causes a larger proximal tubular reabsorption of filtered P resulting in hyperphosphatemia. Lack of PTH action impairs 1,25-(OH)_2_ D production. Low 1,25-(OH)_2_ D contributes to reduced distal tubular Ca reabsorption and intestinal Ca and P absorption, as suggested by animal studies.^(114)^

Conversely, renal complications are found in patients with hypoparathyroidism treated with active vitamin D and/or Ca. Three main complications have been reported: nephrolithiasis, nephrocalcinosis, and chronic kidney disease (CKD).^(115)^ Because the activation of the CaSR inhibits distal tubular Ca reabsorption and enhances urinary Ca excretion, these problems are especially frequent in patients with autosomal dominant hypocalcemia 1 caused by activating mutations in *CASR*.^(116)^ Nephrolithiasis is reported to be a complication in up to 36% of patients with hypoparathyroidism.^(117–121)^ Nephrocalcinosis is reported to complicate the course of hypoparathyroidism in up to 38% of patients, depending on the population and assessment methods.^(118–121)^ Some studies do not allow one to distinguish between nephrolithiasis and nephrocalcinosis, the rate of which ranged from 19% to 31%.^(122–125)^ The rates of CKD range from 2.5% to 41%, depending on definition (estimated GFR lower than 60 mL/min/1.73 m^2^, International Classification of Diseases [ICD] codes or self-report).^(115,117–119,121,122,124–129)^ The risk of CKD progression is higher in patients with chronic hypoparathyroidism.^(130)^ Various factors were found to be associated with the rate of CKD development: age, duration of disease, proportion of time with relative hypercalcemia, number of hypercalcemic episodes, increased Ca × P product, and fractional excretion of P.^(118,122,126,131)^

Other renal manifestations can also complicate the course of hypoparathyroidism, such as acute dehydration during episodes of acute hypercalcemia; however, this has not been clearly reported in the medical literature.

### Skeletal manifestations

**Fig. 3.**
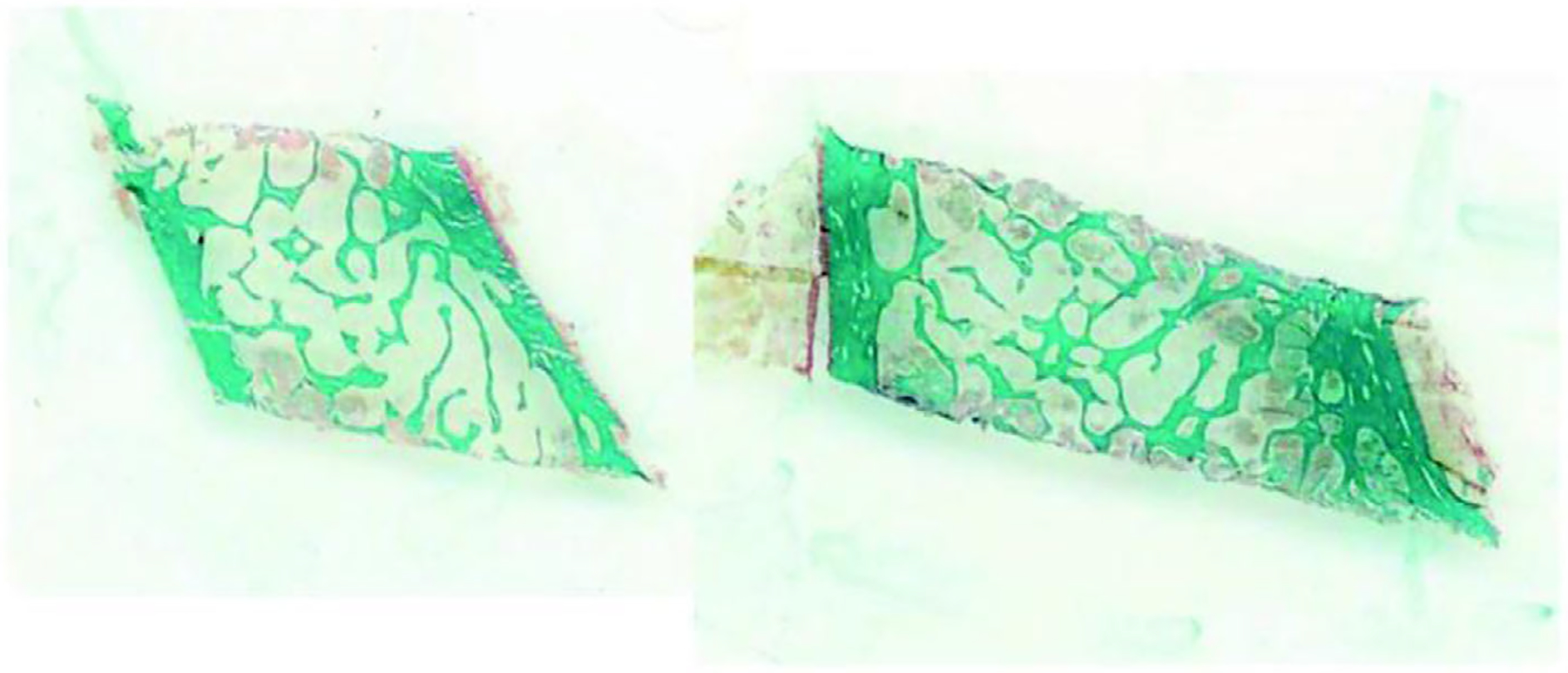
Iliac crest bone biopsies from a control subject (left) and a hypoparathyroid subject (right), Goldner trichrome stain. Note the higher cortical thickness and cancellous bone volume in the hypoparathyroid subject. Reproduced with permission from Rubin and colleagues.^(136)^

**Fig. 4.**
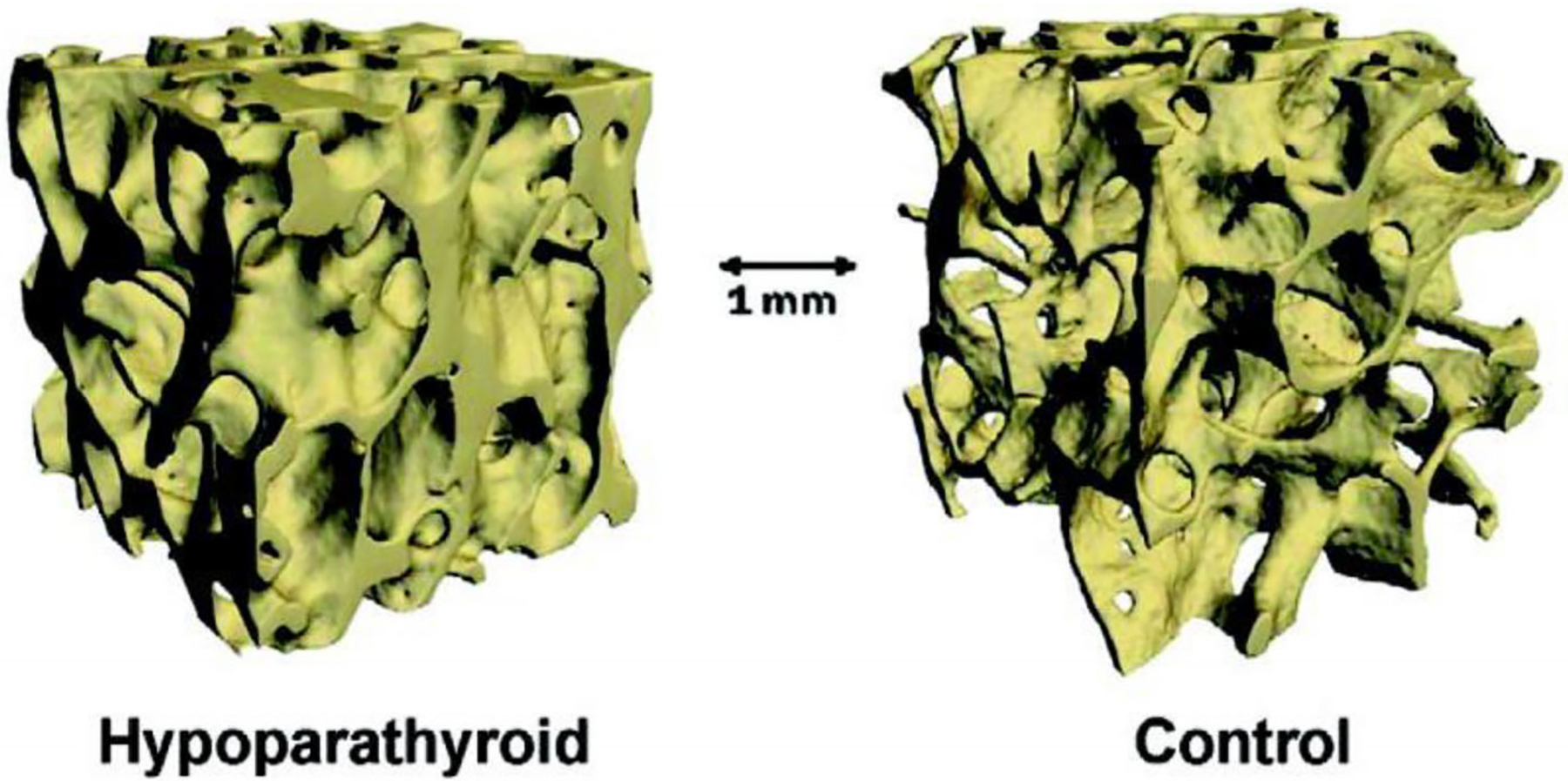
Microcomputed tomographic images of cancellous bone from a hypoparathyroid subject (left) and a control subject (right). Note the higher cancellous bone volume and dense trabecular structure in hypoparathyroidism. Reproduced with permission from Dempster.^(137)^

The principal skeletal manifestation of hypoparathyroidism is a generalized increase in bone mass in both cortical and cancellous compartments. This can be demonstrated by dual-energy X-ray absorptiometry (DXA) and high-resolution peripheral quantitative computed tomography (HR-pQCT).^(132–135)^ Iliac crest bone biopsies in patients with hypoparathyroidism reveal increased cortical thickness and cancellous bone volume (Figs. 3 and 4). The increase in the latter is due to increased trabecular thickness with normal trabecular number and separation.^(136,138)^ Despite the higher bone mass and these structural changes in hypoparathyroidism, the effect on fracture risk in the axial and appendicular skeleton has not yet been firmly established.^(139,140)^

**Fig. 5.**
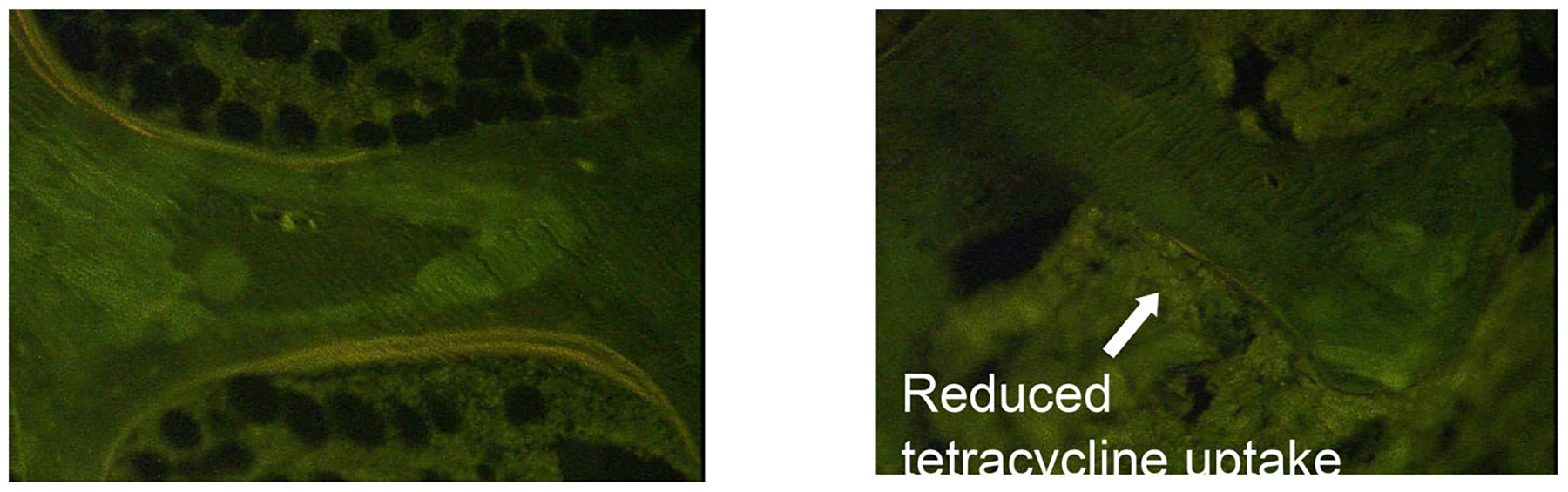
Tetracycline labels in a hypoparathyroid (left) and control subject (right). Note reduction in tetracycline uptake in the hypoparathyroid subject reflecting reduced bone turnover. Reproduced with permission from Rubin and colleagues.^(136)^

**Fig. 6.**
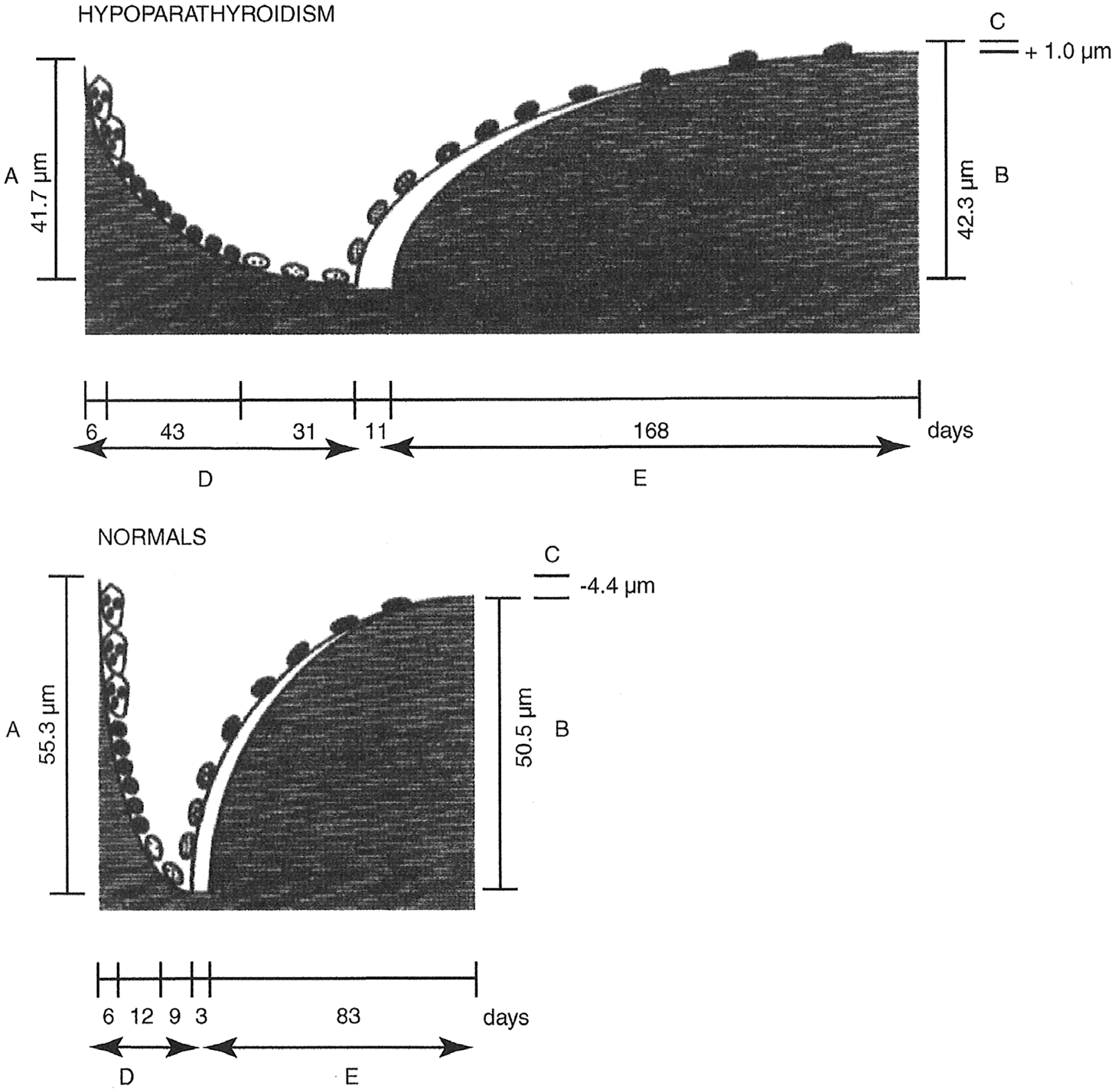
Bone remodeling cycles in hypoparathyroid (upper) and normal (lower) subjects. All phases of the remodeling cycle are elongated in hypoparathyroidism. Reproduced with permission from Langdahl and colleagues.^(142)^

Biochemical markers and bone histomorphometry show that the structural changes in the hypoparathyroid skeleton are associated with a profound reduction in the bone remodeling rate (Fig. 5).^(136,138,141)^ In the first histomorphometric study of bone from hypoparathyroid patients, mineralizing surface, bone formation rate, and remodeling activation frequency were all significantly reduced by 58%, 80%, and 54%, respectively, compared to age- and sex-matched controls.^(142)^ Figure 6 shows the reconstructed remodeling cycles from hypoparathyroid and control subjects in this study. Resorption depth was reduced, and the total resorption period was increased from 26 to 80 days. There was a slightly positive bone balance of approximately 5 μm between the resorption depth and wall thickness of cancellous bone packets in the hypoparathyroid subjects compared to the controls. Thus, slightly more bone was being replaced than was removed in each remodeling cycle. However, this is unlikely to account for the magnitude of the observed increases in bone mass, especially since turnover is so low.

**Fig. 7.**
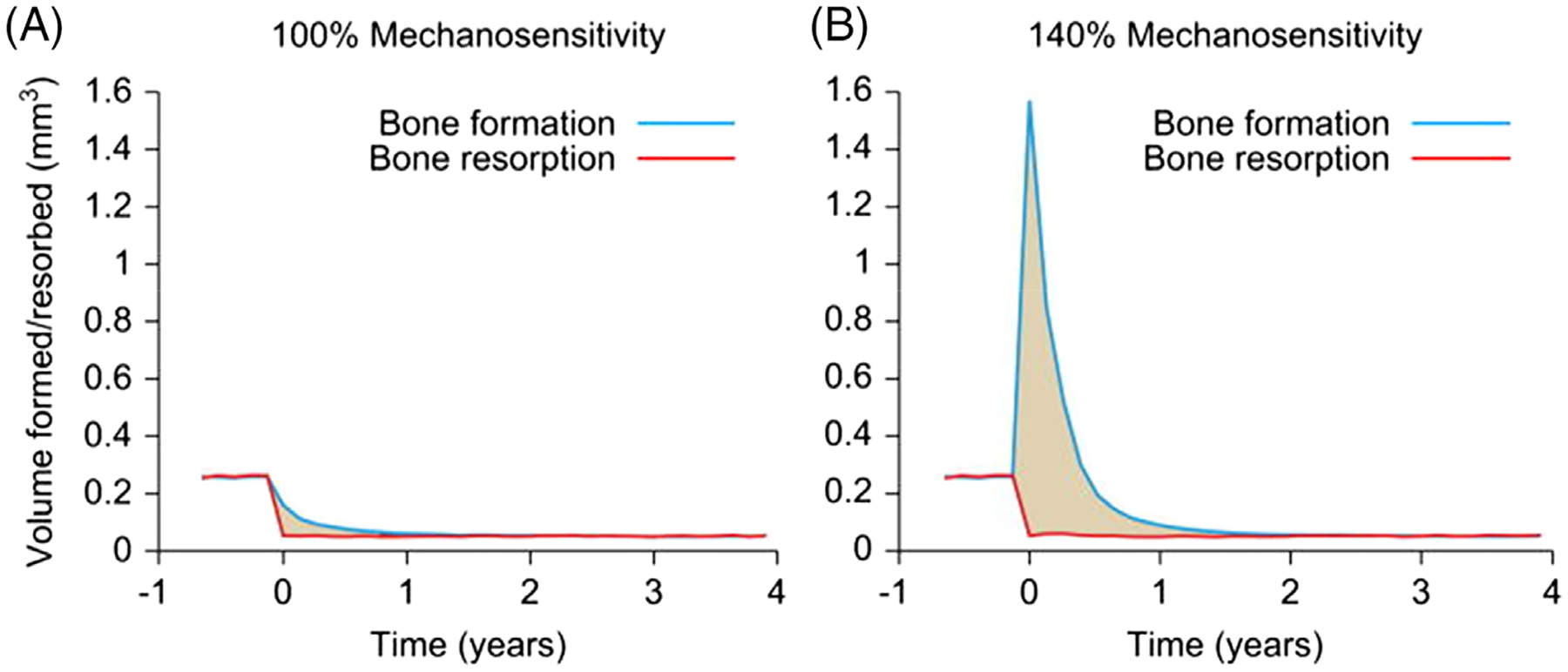
Bone formation and resorption in a simulation of the onset of hypoparathyroidism. In the left panel (*A*), osteocyte mechnaosensitivity is set to 100%. In the panel on the right (*B*), osteocyte mechanosensitivity is set to 140%. As shown in panel (*B*), a net greater increase in bone mass will occur under these conditions. Repoduced with permission from Christen and colleagues.^(143)^

One plausible explanation for the elevated bone mass in hypoparathyroidism has been proposed by Christen and colleagues.^(143)^ The authors used a load adaptive bone modeling and remodeling simulation model to predict changes in microarchitecture due to changes in mechanical loading or cellular activity. The model predicted that, in addition to lowering turnover, the hypoparathyroid state must also cause increased mechanosensitivity of the a normal level of mechanosensitivity (panel a, 100%) osteocytes leading to a marked increase in bone formation during the first year after the onset of the disease (Fig. 7).

### Neurologic, psychiatric, and neuromuscular manifestations

Tetany, muscle stiffness, and seizures are common presenting features in 40% to 60% of patients with hypoparathyroidism.^(144)^ These symptoms recur when calcemic control is disturbed, as with noncompliance with therapy, intercurrent acute infections, or gastritis or diarrhea. Seizures are common in young patients, especially if the etiology of the hypoparathyroidism is nonsurgical. Eighty percent (80%) of the time these seizures are generalized tonic clonic with diffuse slow-wave activity on electroencephalogram, but petit mal, partial, or atonic seizures can also occur.^(145)^ Seizure frequency varies from occasional to daily and has been managed with valproate in 40%, carbamazepine in 27%, and levetiracetam in 13% of cases in a series of 70 patients with idiopathic hypoparathyroidism.^(145)^ Extracellular and intracellular Ca are important for normal neuronal function and muscle contraction. The possible mechanisms underlying the paradox of increased neuromuscular excitability in the hypocalcemic state has been explained by inhibition of CaSRs in brain cells in a hypocalcemic milieu.^(146,147)^ Inhibited CaSRs modulate neuronal “Na leak” and “Ca-activated K” channels. This leads to an inward flow of Na^+^ and a decrease in the outflow of K^+^ in the neurons triggering early depolarization and neuronal excitability. Reduced stimulation of CaSRs in the hypocalcemic state can also release excitatory glutamate neurotransmitter leading to postsynaptic excitability.^(148)^

Basal ganglia calcification (BGC) is a common sign in patients with hypoparathyroidism with a prevalence of 60% to 90%.^(129,144,149)^ Sachs and colleagues^(149)^ observed a 93% prevalence of BGC in patients with hypoparathyroidism when the duration of illness was over two decades. These calcifications are most commonly seen in globus pallidus (69%), followed by putamen (56%), caudate nucleus (55%), gray-white junction (40%), cerebellar parenchyma (31%), thalamus (29%), and dentate nuclei (25%).^(144)^ The cortical gray surface of the brain is spared from calcification. The predisposition of the basal ganglia region for calcification has been explained by a “two-hit mechanism.”^(150,151)^ The “first-hit” is increased expression of several pro-osteogenic molecules (osteonectin, β-catenin, klotho, frizzled-4, ecto-5’-nucleotidase, low-density lipoprotein receptor-related protein 5 [LRP5], Wnt3A, and type 1 collagen) and the presence of neuroprogenitor cells in the basal ganglia.^(151)^ Hyperphosphatemia, resulting from the lack of PTH, constitutes the “second-hit” leading to BGC in hypoparathyroidism. The second-hit acts by decreasing expression of osteoclast carbonic anhydrase-II enzyme and inducing neuro-osteoprogenitor cell differentiation.^(150,151)^ The severity of hyperphosphatemia is also a significant predictor of increases in volume of BGC and spread of calcifications to other intracranial regions when followed over time.^(144)^

Neuropsychological complications are sometimes linked to BGC. Aggarwal and colleagues^(152)^ assessed 62 hypoparathyroid patients and observed extrapyramidal features (mask-like face, rigidity, reduced arm swing, and micrographia) in 15% and cerebellar signs (impaired tandem walk and abnormal heel-shin/finger nose coordination) in 19%. Despite extensive calcification in several patients, Parkinson’s disease requiring levodopa therapy occurred in only two patients. Similarly, cases of chorea, hemiballism, dementia, peripheral neuropathy, and cranial nerve palsies due to raised intracranial pressure can occur, but only occasionally in patients with hypoparathyroidism.^(152)^ Somatic concern (26%), anxiety (47%), guilt (18%), tension (55%), odd mannerism (10%), depressive mood (40%), hostility and suspiciousness (31%) are other manifestations that have been associated with hypoparathyroidism.^(152)^ These neuropsychological dysfunctions do not correlate with the volume of BGC.^(152)^ Rarity of clinical parkinsonism and lack of correlation between cognitive disturbance and BGC could be explained by the presence of relatively intact glucose metabolism and dopaminergic transporters in calcified basal ganglia region in hypoparathyroidism.^(153)^ These observations raise the possibility that mechanical destruction of the surfaces of the corticostriatal tracts where they pass through calcified basal ganglia or alteration in Ca^2+^-dependent enzymes might contribute to the pathogenesis of neuropsychological symptoms in hypoparathyroidism.^(154)^

### Ocular manifestations

Patients with chronic hypoparathyroidism have a twofold to fourfold higher risk of developing cataracts and requiring surgery at an average age of 35 years.^(128,155,156)^ Pohjola^(157)^ reported cataract, papilledema, corneal changes, and loss of eyebrows in 58%, 11%, 10%, and 7% of 118 cases of hypoparathyroidism, respectively. Most cataracts were subcapsular but could be rosette and punctate. Recently, Saha and colleagues^(155)^ described a 46% prevalence of cataracts in 151 cases of idiopathic hypoparathyroidism, which increased to 68% after 8 years of follow-up. Posterior capsular opacification (75%) and decentralization of the lens (25%) are unique long-term complications observed following cataract surgery in patients with idiopathic hypoparathyroidism.^(155)^ Cataracts are associated with intracranial calcification, but not with nephrocalcinosis, suggesting different mechanisms for calcification.^(131)^ The mechanisms of cataract development in hypoparathyroidism are not clear but could involve the hypocalcemic environment per se. Cataracts can be induced in an experimental model in vitamin D–deficient rats.^(158)^ In this model, reduction of s-Ca led to decreased Ca content of the aqueous humor and increased Na content of the lens. Clark^(159)^ showed decreased deposition of Ca in the lens extracted from pigs, after they were immersed in CaCl_2_ solution along with PTH. Therefore, cataracts in hypoparathyroidism could also possibly be due to lack of PTH leading to increased deposition of Ca in the lens.^(159)^

### Quality of life

Chronic hypoparathyroidism negatively impacts quality of life (QOL).^(120,127,160–163)^ Patients and caregivers carry a substantial burden of illness.^(164,165)^ Impact on QOL has been assessed in cohorts around the world that include postsurgical patients^(120,127,160–163)^ and patients with nonsurgical hypoparathyroidism.^(127,152,161)^ Detailed review of QOL studies in patients with hypoparathyroidism is included in elsewhere.^(21)^

### Future directions, unanswered questions, and research agenda

There are several unanswered questions, and further studies are necessary.

For example, hypocalcemia has been reported in patients with viral infection with such as severe acute respiratory syndrome-coronavirus-2 (SARS-CoV-2), SARS-CoV, and *Ebola-virus*.^(166)^ However, the mechanism and the natural course of this hypocalcemia has not been well characterized.

The mechanism underlying the profound increase in bone mass in hypoparathyroidism is not clearly understood.

PTH treatment improves several symptoms and QOL in patients with hypoparathyroidism.^(167–170)^ However, it is not completely understood whether this improvement is caused by direct actions of PTH on various tissues or by a better control of s-Ca levels. It is not clear what the optimal s-Ca levels are for a given patient, because some patients complain of symptoms even when s-Ca is normal. It is possible that the lack of PTH accounts for such problems.

Many etiologies for hypoparathyroidism have been identified, but the pathogenesis is unexplained in a large number of patients. The term idiopathic or primary hypoparathyroidism is still used in the literature because it is difficult to determine the etiologies in all cases of hypoparathyroidism. Future research should address as yet unknown causes of this disease.

The value of emerging technologies and intraoperative assessment of parathyroid viability on the functionality of the parathyroid glands after surgical manipulation merits further assessment.

## Figures and Tables

**Table 1.** Etiology of Hypoparathyroidism

Postsurgical
Autoimmune
Autoimmune polyglandular syndrome-1
Isolated autoimmune hypoparathyroidism
Infiltrative
Granulomatous disease
Sarcoidosis
Amyloidosis
Metastatic cancer
Riedel struma
HIV
Destructive
Radiation—external beam and ionizing radiation
Metal deposition
Wilson’s disease (copper)
Hemochromatosis (iron)
Transfusion dependence (iron)
Aluminum toxicity
Metabolic causes
Hypermagnesemia
Magnesium deficiency
Burn injury
Fetal exposure to retinoids, alcohol or hyperglycemia or maternal hypercalcemia
Toxic agents^a^
Genetic disorders of isolated or syndromic hypoparathyroidism^b^
Mitochondrial disorders^c^
Idiopathic^d^

a Asparaginase, Ethiofos, immune checkpoint inhibitors (eg, nivolumab).

b Isolated parathyroid hypoplasia or PTH hyposecretion including mutations in *PTH*, *CASR*, *GNA11*; DiGeorge Sequence; *TBX*; CHARGE syndrome; Hypoparathyroidism, Deafness, Renal anomaly (HDR) syndrome (Barakat syndrome); Sanjad-Sakati syndrome, and Kenny-Caffey syndrome; Smith-Lemli-Opitz syndrome.

c Kearns-Sayre syndrome; Pearson Marrow-Pancreas syndrome; MELAS syndrome; Long-chain 3-hydroxy-acyl-coenzyme A dehydrogenase (LCHAD) deficiency; medium-chain acyl-CoA dehydrogenase deficiency (MCADD).

d No etiology determined after extensive workup completed.

**Table 2.** Subclassification of Permanent Surgical Hypoparathyroidism

Subcategory of permanent surgical hypoparathyroidism	Biochemical diagnosis	Treatment
Hypoparathyroidism	Low or undetectable iPTH Hypocalcemia +/− High serum phosphorus	Elemental calcium 2 to 4 g/day or more^a^ Calcitriol 0.5 to 2 μg/day or more^a^ PTH replacement^b^ Thiazide diuretic
Parathyroid insufficiency	Low iPTH Elevated serum phosphorus Normal or only slightly reduced serum calcium	Elemental calcium titrated Maintain vitamin D sufficiency with vitamin D3 or D2
Relative parathyroid insufficiency	Normal iPTH level but insufficient to maintain serum phosphorus and/or calcium within normal limits	Address any impaired absorption issues or drugs that lower serum calcium^c^ Maintain serum calcium levels with elemental calcium supplements

Abbreviation: iPTH = intact parathyroid hormone.

a These are average calcium and calcitriol doses and can be higher and lower depending on individual patient needs.

b Available as recombinant human PTH^(1–4,6–84)^ in certain countries.

c Malabsorption syndromes and diseases, short gut, gastric bypass, bisphosphonate or denosumab therapy. Minimize use of thiazide diuretics and/or proton-pump inhibitors. All of these mitigating issues can make chronic hypoparathyroidism worse.

**Table 3.** Symptoms of Acute Hypocalcemia Postoperatively^a^

Mild	Severe
Neuromuscular	
Chvostek’s sign (facial nerve excitability)	Confusion
Trousseau’s sign (carpal spasm)	Seizures
Paresthesia (perioral and extremity)	Tetany
Muscle cramps	Laryngospasm
Headache	Bronchospasm
Cardiac	
Prolonged QTc interval	Congestive heart failure
T-wave inversion	Ventricular tachycardia
	Torsades de pointes Other arrhythmias

a These signs and symptoms noted may be present during hypocalcemic episodes in the patient with chronic hypocalcemia or hypoparathyroidism of any etiology.

**Table 4.** Risk Factors for Postsurgical Hypocalcemia

Factors	Risk of hypoparathyroidism or incidental parathyroidectomy	Highest level of evidence
Patient factors		
Obesity BMI >40 kg/m^2^	OR 1.94^(37,38)^	National registry multivariate analysis (*n* = 8381)
Vitamin D deficiency	RR 1.92–2.45^(85)^	Meta-analysis of 39 prospective/retrospective studies
Pediatric	7.3% to 22%^(42,43,86)^ CLND OR 16.18^(42,45)^	National registry (*n* = 740)
Disease factors		
Graves’ disease	Transient rate 70.5%, permanent rate 27.3%. OR 1.75 to 4.40^(6,12,87)^	Meta-analysis of 115 observational studies
Malignancy	RR 1.60^(39)^	Meta-analysis 35 retrospective studies
Concomitant thyroid/parathyroid surgery	OR 2.38 to 7.23^(88)^	Single institution (*n* = 1065)
Operative factors		
Central lymph node Level VI dissection	OR 1.48 to 4.44^(10,36,38,41)^	Prospectively maintained national registry (*n* = 8672)
Reoperative surgery	OR 1.44^(41)^	Prospectively maintained national registry (*n* = 8672)
Transoral approach	Transient OR 0.96, permanent OR 0.32^(89)^	Meta-analysis 6 retrospective studies
Surgical time >3 hours	OR 2.63^(37)^	National registry multivariate analysis (*n* = 8381)
Low surgeon volume	OR 2.94^(36)^	Single center (*n* = 1114)
Incidental parathyroidectomy	12.4%^(39)^	Meta-analysis 35 cohort studies

## Data Availability

The data that support the findings in this study are openly available in PubMed, MEDLINE, EMBASE, and the Cochrane databases.
